# Recurrent peri‐myocarditis: A rarer but unfortunate redundant presentation of thyroid storm

**DOI:** 10.1002/ccr3.8533

**Published:** 2024-02-13

**Authors:** K. C. Nabin, Pratik Bhattarai, Erum Zahid, Muhammad Nabeel Pasha

**Affiliations:** ^1^ Pulmonary and Critical Care Medicine One Brooklyn Health Brooklyn USA; ^2^ Pulmonary and Critical Care Medicine Brookdale University Hospital Medical Center Brooklyn USA; ^3^ Pulmonary and Critical Care Medicine Interfaith Medical Center Brooklyn USA; ^4^ Oxford University Clinical Research Unit Nepal Lalitpur Nepal; ^5^ Medicine and Surgery Manipal College of Medical Science Pokhara Nepal; ^6^ Pulmonary, Critical care and Sleep Medicine Brookdale University Hospital and Medical Center Brooklyn USA; ^7^ Internal Medicine Interfaith Medical Center Brooklyn USA

**Keywords:** cardiomyopathy, myocarditis, thyroid storm, thyrotoxicosis

## Abstract

Thyroid storm represents a critical and life‐threatening complication from hyperthyroidism, with a notable mortality risk. Limited literature reports have explored the correlation between thyroid storm and peri‐myocarditis, although the precise pathophysiological underpinnings remain unclear. The pathophysiology of how thyroid storm and peri‐myocarditis are associated is not clearly understood; however, unfavorable prognostic factors include atrial fibrillation and recurrent thyrotoxicosis. Here, we present a case concerning recurrent peri‐myocarditis concomitant with a thyroid storm.

## 1 INTRODUCTION

Thyroid storm is an acute and life‐threatening complication of hyperthyroidism with a significant mortality rate, and about 5% of them have initial heart failure presentation, while others may have cardiac presentations like tachycardia and atrial fibrillation.^1^ A few cases are reported in the literature on thyroid storm and its association with peri‐myocarditis. The exact pathophysiology of how thyroid storm and peri‐myocarditis are associated is not clearly understood; however, unfavorable prognostic factors include atrial fibrillation and recurrent thyrotoxicosis.^2^ We have discussed a case of recurrent peri‐myocarditis with a thyroid storm.

## 2 CASE PRESENTATION

A 47‐year‐old male was brought into the emergency department with poor oral intake and generalized weakness with a past medical history significant for schizophrenia and hyperthyroidism due to Grave's disease. The patient was tachycardic with a heart rate of 180 bpm and was afebrile. Upon thorough clinical examinations, laboratory results, and investigations, we suspected the patient was having a thyroid storm and was admitted to the intensive care unit (ICU). Records showed the patient had a similar admission 4 months prior when the patient presented with catatonic symptoms. During the same visit, his cardiac MRI (magnetic resonance imaging) showed the features of myocarditis; electrocardiogram (EKG) showed—normal sinus rhythm, bilateral atrial enlargement, and left anterior fascicular block. A transthoracic echocardiogram (TTE) was done on the same admission and was found to have reduced systolic function with diffuse hypokinesis.

## 3 INVESTIGATIONS AND TREATMENT

**FIGURE 1:**
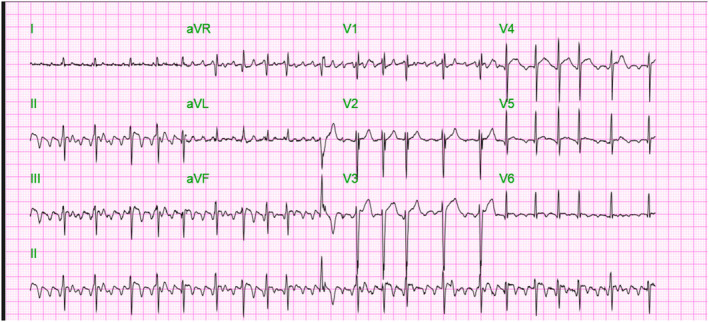
Electrocardiogram (EKG) showing atrial fibrillation with rapid ventricular rhythm (RVR).

Given the patient's presentation with tachycardia, we obtained an initial EKG that revealed atrial fibrillation with rapid ventricular rate (RVR) (Figure 1), which responded to Cardizem infusion. Labs (Table 1) were remarkable for elevated troponin I, leukocytosis with elevated white blood cell, increased creatinine (with a prior level of 0.5), thyroid stimulating hormone (TSH) <0.015, and Free T4 >6.99, free T3 >651 consistent with thyrotoxicosis. An initial Burch‐Warsofsky point scale of 50 points was suggestive of a thyroid storm and admitted to the ICU. The patient was managed with propranolol, propylthiouracil (PTU), Lugol's iodine, and hydrocortisone.

**TABLE 1:** Remarkable laboratory results and trends.

Tests	Results	Reference range with units
On Admission‐ Pretreatment		
TSH	<0.015	0.3–4 mU/L
fT4	>6.99	1.4–1.77 ng/dL
fT3	>651	3.8–4.4 pg/mL
Troponin I	0.505	<0.04 ng/mL
WBC	16.3 k	4–11 *103/cu mm
Creatinine	1.99	0.7 to 1.3 mg/dL
On Day 2‐posttreatment		
TSH	<0.015	0.3–4 mU/L
fT4	>6.99	1.4–1.77 ng/dL
fT3	>651	3.8–4.4 pg/mL
AST	>1500	14–36 U/L
ALT	>2500	0–35 U/L
Post plasmapheresis		
TSH	0.022	0.3–4 mU/L
fT4	2.46	1.4–1.77 ng/dL
fT3	2.3	3.8–4.4 pg/mL
AST	682	14–36 U/L
ALT	641	0–35 U/L
Urea	69	5–20 mg/dL
Creatinine	0.8	0.7–1.3 mg/dL

Abbreviations: ALT, alanine transaminase; AST, aspartate aminotransferase; fT3, free triiodothyronine; fT4, free thyroxine; TSH, Thyroid stimulating hormone; WBC, White blood cell.

**FIGURE 2:**
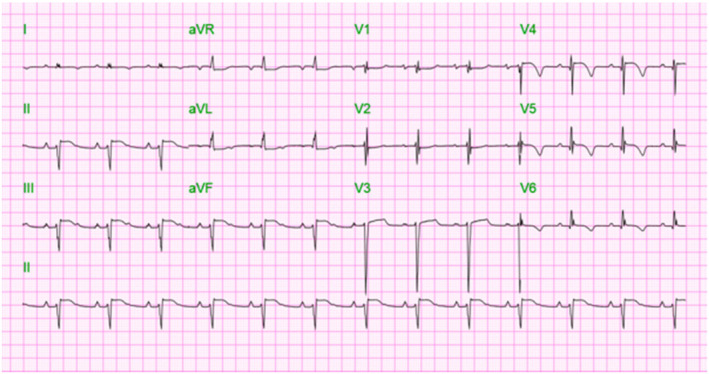
Electrocardiogram (EKG) showing ST segment elevation on leads II, III aVF and V3, V4.

The patient progressively deteriorated in his mental status, requiring intubation to protect his airways, and went into shock (Blood Pressure 77/53 mmHg), requiring vasopressor and inotropes. Troponin I (TrI) gradually trended up (peak TrI 23.60) with changes in EKG concerning ST elevation myocardial infarction (STEMI) in anterior and inferior leads (Figure 2). An echocardiogram revealed severely reduced systolic function with an ejection fraction of 10%–15% and severe diffuse hypokinesis. Left heart cardiac catheterization revealed no angiographically significant coronary artery disease and left ventricular end‐diastolic pressure (LVEDP) ~ 15 mm/Hg. The patient was started on guideline‐directed medical therapy (GDMT) for non‐ischemic cardiomyopathy secondary to thyrotoxicosis.

**FIGURE 3:**
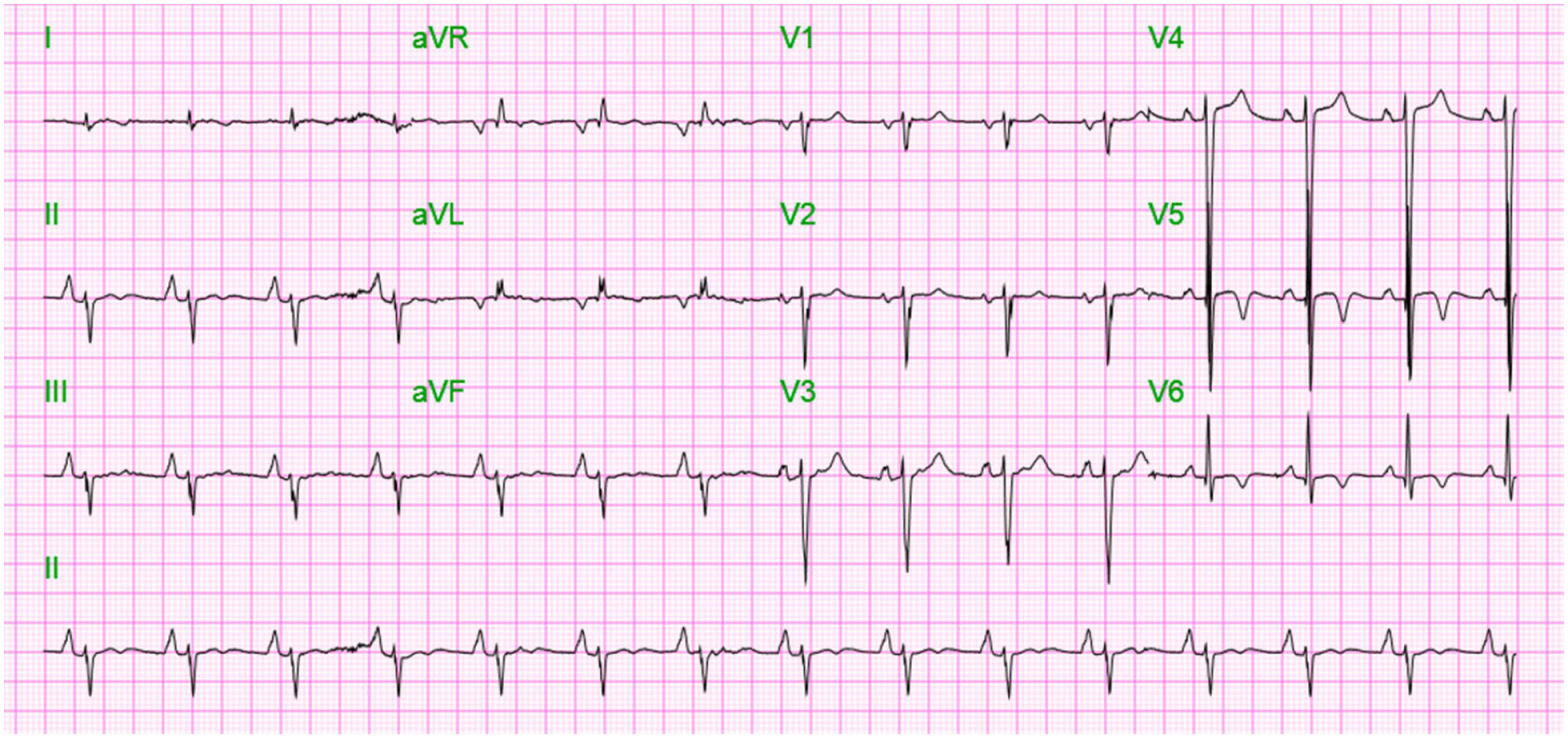
Electrocardiogram (EKG) showing normal sinus rhythm post plasmapheresis.

On the second day of hospitalization, his course got complicated with abnormal liver function (Table 1) suggestive of shock liver with AST and ALT levels above 1500 and 2500, respectively and TSH <0.015, and free T4 >6.99, free T3 >651. The patient was started on N‐acetyl cysteine (NAC), and PTU was discontinued. The patient had a session of plasmapheresis with target‐free T4 <2.5. Following the first plasmapheresis session, there was an improvement in liver function, renal function, and thyroid hormones (Table 1), and EKG showed normal sinus rhythm (Figure 3), meeting initial goals. The patient failed to wean off the ventilator in the ICU; eventually, a tracheostomy was performed, and he was transferred to the medical floor with a plan to be discharged to subacute rehabilitation for chronic weaning off the ventilator.

**FIGURE 4:**
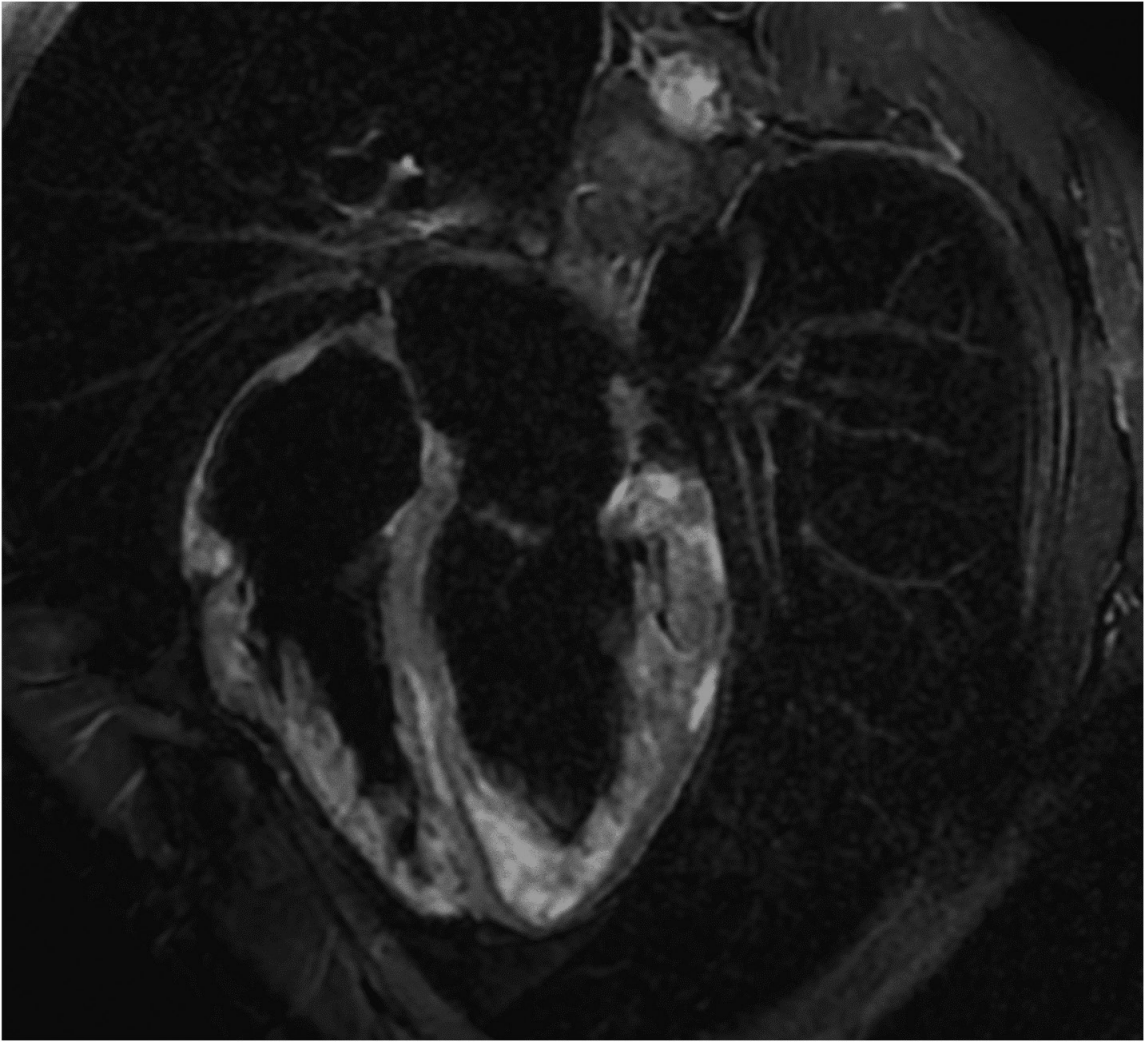
Cardiac MRI with gadolinium showing high T2 signal and late gadolinium enhancement in the pericardial region of basal inferolateral/inferior and all mid segments, consistent with non‐ischemic cardiac myopathy.

During this admission, a cardiac MRI (Figure 4) was done, and it revealed Left Ventricular dilation with mildly decreased systolic function and left ventricular ejection fraction (LVEF) of 43%. Focal mild hypokinesis, high T2 signal, and late gadolinium enhancement in this of the pericardial region of basal inferolateral/inferior and all mid segments, consistent with non‐ischemic cardiac myopathy, likely peri‐myocarditis.

## 4 DISCUSSION

Thyrotoxicosis is a clinical condition characterized by persistently elevated levels of FT3, FT4, or both, which correlate with increased or reduced thyroid metabolic activity.^3^ Thyroid storm, also known as thyrotoxic crisis, is the most severe form of thyrotoxicosis and is typically characterized by organ malfunction and failure.^4^ Cardiovascular manifestations are the most severe presentation of thyrotoxicosis, most commonly atrial fibrillation and tachycardia‐induced cardiomyopathy also, coronary vasospasm, and autoimmune myocarditis, all of which can mimic ST‐segment elevation myocardial infarction (STEMI).^1^, ^5^ Excess thyroid hormone levels have also been independently associated with coronary events. A study by Peters et al. found a strong association between excess T3 and coronary events such as angina pectoris or myocardial infarction (MI).^6^ In this case, we reported non‐ischemic cardiomyopathy, likely peri myocarditis, as an uncommon complication of a thyroid storm.

Patients with signs and symptoms of acute Myocardial Infarction (MI) in the setting of hyperthyroidism but normal coronary arteries on angiography should also be suspected of having coronary vasospasm.^1^ A review article discovered that six out of 21 patients with thyrotoxicosis‐related acute MI had signs of hyperthyroidism. Normal coronary angiography was reported in 13 of the 21 patients, and coronary vasospasm without thrombus blockage was found in three.^7^

Graves' disease is a well‐known autoimmune thyroid disease with a rare cause of acute perimyocarditis. The mechanism of Graves' disease‐associated perimyocarditis is unknown. In one study, Mavrogeni et al. investigated the association between autoimmune thyroid disease and myocarditis in 250 individuals with hyperthyroidism and persistently elevated antithyroglobulin and anti‐microsomal antibodies despite being euthyroid on treatment. Out of 250, only 50 individuals experienced ongoing cardiac symptoms such as chest discomfort, dyspnea, and palpitations. Most underwent cardiac magnetic resonance imaging (CMR), which showed myocarditis. In selected cases, endomyocardial biopsies revealed lymphocytic infiltration without viral infection, indicating an autoimmune origin.^8^ These infiltrates have previously been observed in individuals with autoimmune myopericarditis caused by hyperthyroidism, supporting autoimmunity as the underlying mechanism.^8^, ^9^ Our case report, on the other hand, involves a patient with active hyperthyroidism symptoms, high thyroxine, suppressed TSH, and cardiac symptoms with elevated cardiac enzymes, which differs from the study done by Mavrogeni et al. given that the patient was clinically euthyroid.

This report presents a case of recurrent perimyocarditis caused by a thyroid storm. Our patient was a known case of hyperthyroidism (Grave's disease). He was diagnosed as having a thyroid storm and managed with propranolol, propylthiouracil (PTU), Lugol's iodine, and hydrocortisone. Later in the course, he developed ST elevations, which could be caused by coronary vasospasm, autoimmune myocarditis, or cardiomyopathy. The lack of reciprocal ST depressions on ECG and specific wall‐motion abnormalities on echocardiography suggests a different process than coronary thrombosis. Furthermore, his coronary angiography revealed no signs of coronary stenosis. Moreover, the involvement of the myocardium, as seen by elevated cardiac biomarkers, as well as generalized hypokinesis with decreased ventricular function, supported the development of peri myocarditis.

## 5 CONCLUSION

Thyroid storm can present as acute peri‐myocarditis requiring thyroid function evaluation during such presentation. Compliance with antithyroid therapy and follow‐up on cardiac function will possibly ascertain the prevention of the recurrence of such fatal complications.

## AUTHOR CONTRIBUTIONS

**K. C. Nabin:** Conceptualization; data curation; supervision; writing – original draft; writing – review and editing. **Pratik Bhattarai:** Supervision; writing – original draft; writing – review and editing. **Erum Zahid:** Conceptualization; supervision; writing – original draft; writing – review and editing. **Muhammad Nabeel Pasha:** Writing – original draft; writing – review and editing.

## FUNDING INFORMATION

No source of funding.

## CONFLICT OF INTEREST STATEMENT

There is no conflict of interest to be declared.

## ETHICAL APPROVAL

Not Applicable.

## CONSENT STATEMENT

Written informed consent was obtained from the patient for publication of this case report and accompanying images. A copy of the written consent is available for review by the Editor‐in‐Chief of this journal on request.

## Data Availability

All data underlying the results are available as part of the article, and no additional source data are required.
